# Resurgence of Pertussis Infections in Shandong, China: Space-Time Cluster and Trend Analysis

**DOI:** 10.4269/ajtmh.19-0013

**Published:** 2019-04-15

**Authors:** Yuzhou Zhang, Hilary Bambrick, Kerrie Mengersen, Shilu Tong, Lei Feng, Li Zhang, Guifang Liu, Aiqiang Xu, Wenbiao Hu

**Affiliations:** 1 School of Public Health and Social Work, Institute of Health and Biomedical Innovation, Queensland University of Technology, Brisbane, Australia;; 2 School of Mathematical Sciences, Queensland University of Technology, Brisbane, Australia;; 3 School of Public Health, Institute of Environment and Human Health, Anhui Medical University, Hefei, China;; 4 Shanghai Children’s Medical Centre, Shanghai Jiao-Tong University, Shanghai, China;; 5 Shandong Provincial Centre of Disease Control and Prevention, Jinan, China

## Abstract

Although vaccination is effective in preventing infection, pertussis remains endemic worldwide, including China. To lead better targeted prevention strategies, we examined dynamics of spatial and temporal patterns of pertussis transmission in Shandong, China, from 2009 to 2017. We used space-time cluster analysis, logistic regression analysis, and regression tree model to detect the changes in spatial patterns of pertussis infections in Shandong Province, China, between periods (2009–2011, 2012–2014, and 2015–2017). The yearly pertussis incidence rates dramatically increased by 16.8 times from 2009 to 2017. Shifting patterns of peaks of pertussis infections were observed over both time (from June–July to August–September) and space (from Linyi to Jinan), with increasing RR from 4.1 (95% CI: 2.3–7.4) (2009–2011) to 6.1 (95% CI: 5.6–6.7) (2015–2017) and obvious coincidence of peak time. West Shandong had larger odds of increased infections over the study period (odds ratio: 1.52 [95% CI: 1.05–2.17]), and pertussis had larger odds of spreading to east (odds ratio: 2.32 [95% CI: 1.63–3.31]) and north (odds ratio: 1.69 [95% CI: 1.06–2.99]) over time. Regression tree model indicated that the mean difference in yearly average pertussis incidence between 2009–2011 and 2015–2017 increased by more than 4-fold when the longitudes of counties are < 118.0°E. The geographic expansion of pertussis infection may increase the risk of epidemic peaks, coinciding with increased infections in the future. The findings might offer evidence for targeting preventive measures to the areas most in need to minimize the impact of the disease.

## INTRODUCTION

Pertussis (also known as whooping cough or 100-day cough) caused by *Bordetella pertussis* is a highly contagious respiratory disease that imposes a substantial global health burden. Despite extensive immunization programs, pertussis is still endemic throughout the world, with approximately 50 million cases and 297,000–409,000 related deaths worldwide each year.^1,2^ The disease has resurged in many countries over recent years, such as the United States, Japan, and Australia.^3–5^

In China, a vaccination program against pertussis was introduced in the early 1960s, when three doses of whole-cell vaccine combined with diphtheria and tetanus toxoids (DTwP) were given at 3, 4, and 5 months of age. A booster dose at 18–24 months of age was added in 1978.^6^ The DTwP vaccine was replaced by a diphtheria and tetanus toxoids (DTaP) vaccine from May 2007.^7^ In addition, there was an increase in the vaccination coverage for target populations from 58% to 99% during the period 1983 through 2018.^8^ However, despite these efforts, China has experienced a dramatic increase in pertussis occurrences over the last decade.^7,9,10^ In addition, the appearance of macrolide resistance of *B. pertussis* and relatively inadequate levels of anti-pertussis toxin IgG among populations has become current public health concerns in Shandong.^11,12^

Strong seasonal patterns can be found for many infectious diseases, as climatic factors can affect both the survival of the pathogen in the environment and human population behaviors.^13^ Interestingly, the seasonal patterns of pertussis are variable in different countries. Previous studies have reported that the annual peak incidence of pertussis was in summer in the Netherlands,^14^ but in autumn and winter in Australia,^15^ and spring in Canada.^16^ Moreover, the seasonal patterns of pertussis epidemics can even change in the same area for different time periods.^16,17^ This indicates that further research is needed to qualify the seasonality of pertussis by different areas and time periods.

Advances in spatial analysis enable health authorities and governments to garner insights about the patterns and spread of infectious diseases, high-risk areas and spatial clusters, and trends over time. These insights in turn may be used to improve disease monitoring, develop more specific early warning systems, and more efficiently target preventative measures to minimize the impact of diseases and reduce morbidity and mortality.^18,19^

Present knowledge about the transmission patterns of pertussis in China is limited, especially the spatial and temporal dynamics of the transmission over time. This study aims to examine the temporal patterns and spatial variation of pertussis notifications in Shandong Province, China, between 2009 and 2017 to identify the dynamical high-risk areas of infection and provide a deeper understanding of the epidemiological characteristics of this disease.

## METHODS

### Study site and data collection.

**Figure 1.**
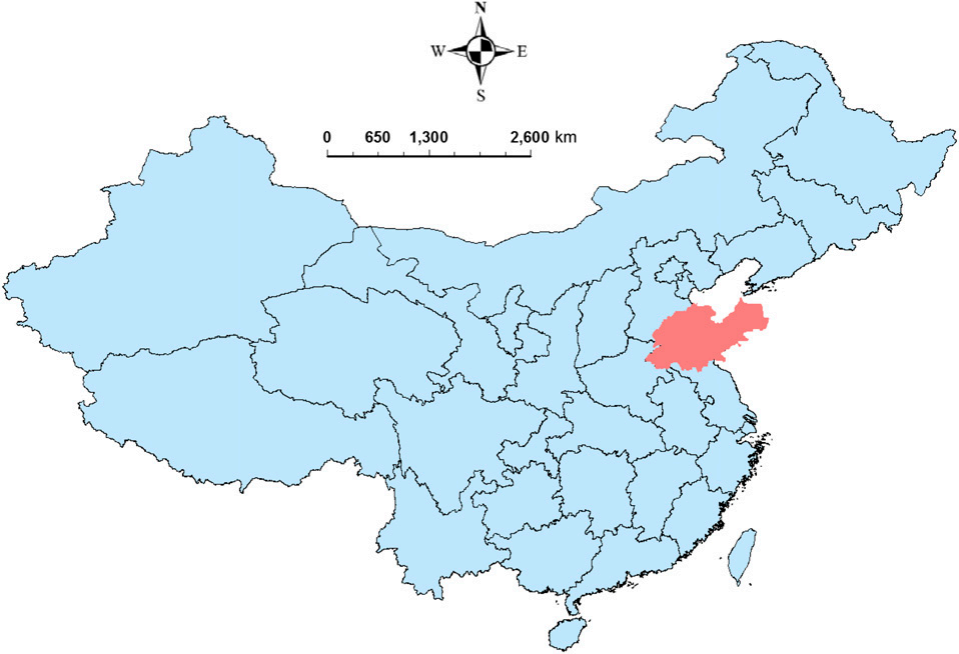
The location of Shandong Province, China. This figure appears in color at www.ajtmh.org.

Shandong Province is located in the east of China (Figure 1). It is the second largest province by population in China, with nearly 96 million people and 137 counties.^20^ Shandong has a temperate climate with four distinct seasons, lying in the transition between the humid subtropical and humid continental zones.^21^ Average annual temperatures are 11–14°C (–5 to 1°C in January and 24–28°C in July).^21^ The mean values of the population size and population density (population/km^2^) at the county level were 685,204 (range: 44,025–1,603,659) and 1,018 (range: 104–18,156), respectively.^20^ The pertussis vaccine coverage in 2011 was more than 95% in Shandong Province.^22^

Weekly data on the total number of clinically and laboratory-confirmed pertussis cases for each county in Shandong Province were retrieved for the period of January 2009 to December 2017 from the Chinese National Notifiable Disease Reporting System (CNNDRS), which is widely used in previous studies.^9,10^ The detailed diagnosis confirmation of pertussis (WS 274-2007) was issued by the Chinese Ministry of Health on April 17, 2007, and all laboratory-confirmed and clinically diagnosed cases must be reported into CNNDRS within 24 hours after diagnosis.^23^ Annual population data at the county level during the study period were collected from the Shandong Provincial Bureau of Statistics (http://www.stats-sd.gov.cn/).

### Data analysis.

#### The assessment of temporal patterns.

Time series decomposition procedures were performed to describe the seasonal factors of pertussis epidemics in the study period.^15^ This method decomposes time series data in three (trend, systematic seasonal factors, and residual) components using a sequence of smoothing operations.^15,24,25^ As pertussis has an epidemic peak every 3–5 years,^26^ the study period (2009–2017) was divided into three subsets, including period 1 (2009–2011), period 2 (2012–2014), and period 3 (2015–2017), to compare the seasonality of infections in different time periods. We used cumulative notifications from the same week of each research period to discover the weekly variation of pertussis notifications between research periods. Moreover, we transformed the weekly pertussis data to seasonal cumulative notifications (spring, summer, autumn, and winter) to compare the seasonality of pertussis epidemics by the four seasons in different periods. Seasonal decomposition procedures were also performed to compare systematic seasonal variations of pertussis infections for different periods. More specifically, we used boxplots and heat maps to summarize the results at the provincial and county levels, respectively. These analyses were carried out using SPSS Statistics software, version 25 (SPSS, Inc., Chicago, IL) and R software (version 3.5.1; R Development Core Team, Boston, MA).

#### Space-time cluster analysis.

High-risk spatial clusters of pertussis infections for each period were identified using SaTScan software (version 9.6; Martin Kulldorff, Boston, MA). For this purpose, a purely spatial model was fitted assuming a Poisson distribution for the number of pertussis notifications, with the RR moderated by the respective population. This approach creates a (spherical) window in space, and then compares the numbers of expected and observed cases in and outside of the spatial window.^27,28^ The spatial locations were determined by the longitude and latitude of the centroid of each county. We used annual notifications and annual mean populations for each county and each period in the analysis. The maximum size of cluster search windows in our model was set as 50% of the population size.^29,30^ RR for disease clustering was also calculated, and the definition of RR is given in the Supplemental Material.^31^

#### Analysis of temporal and geographic patterns in epidemic dynamics.

Several indicators have been used to measure the magnitude and severity of disease epidemics, with the annual incidence rate being the most common indicator in measuring the temporal risk of an epidemic.^32^ Thus, we transformed the weekly notifications to the yearly mean incidence rate of pertussis (cases/100,000 population) by period, and then mapped the mean annual incidence rate at the county level for each period to discover the spatial patterns of pertussis epidemics. In addition, to discover the transmission dynamics in both space and time, the difference in incidence rates between each subset of period was mapped at the county level. We also examined the geographic expansion of transmission through mapping the affected areas by period.

#### Spatial dispersion of pertussis infections.

To examine whether there were spatial trends in the differences of pertussis incidences and the geographic expansion of pertussis infections between periods, we fitted binary and multinomial logistic regression to determine the natural logarithm of the differences and the expansion in the distribution of pertussis cases on the locations (latitude and longitude) of counties. We transformed the differences of incidences and the infection expansion to two groups (0 [decreasing or no differences] and 1 [increasing]) and three groups (0 [never affected], 2 [periods 2 and 3 only], and 3 [period 3 only]) separately. These analyses were conducted using the data of subsequent period compared with that of previous period. The analysis was carried out using SPSS Statistics software, version 25 (SPSS, Inc.).

To avoid the parametric associations of the models described previously, a regression tree model was also used to divide the locations (latitudes and longitudes) of counties into subsets that were homogeneous with respect to the differences in incidences rates for each period. This approach has the advantage of predicting the relationship between a response variable and one or more exploratory variables by constructing a flexible, robust, nonparametric model.^33^ Cross-validation was used to select the tree size using estimated prediction errors. The best model is defined as having the smallest tree size and an estimated error rate within one standard error of the minimum.^34^ This analysis was performed using the “rpart” package of R version 3.5.1.

## RESULTS

### Descriptive analysis.

**Figure 2.**
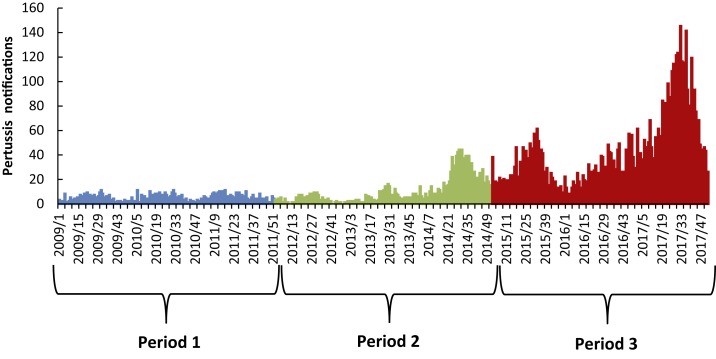
The epidemic curve of weekly pertussis infections in Shandong Province, 2009–2017. This figure appears in color at www.ajtmh.org.

The weekly trend in pertussis occurrences at the provincial level is shown in Figure 2. During 2009–2017, there is an apparent increasing trend in pertussis notifications; the mean of weekly notifications was 20.3 (range: 0–145), but this increased dramatically over time from 4.9 in period 1 (range: 0–11) to 41.6 in period 3 (range: 6–145).

### The temporal variation in seasonality of pertussis infection.

**Figure 3.**
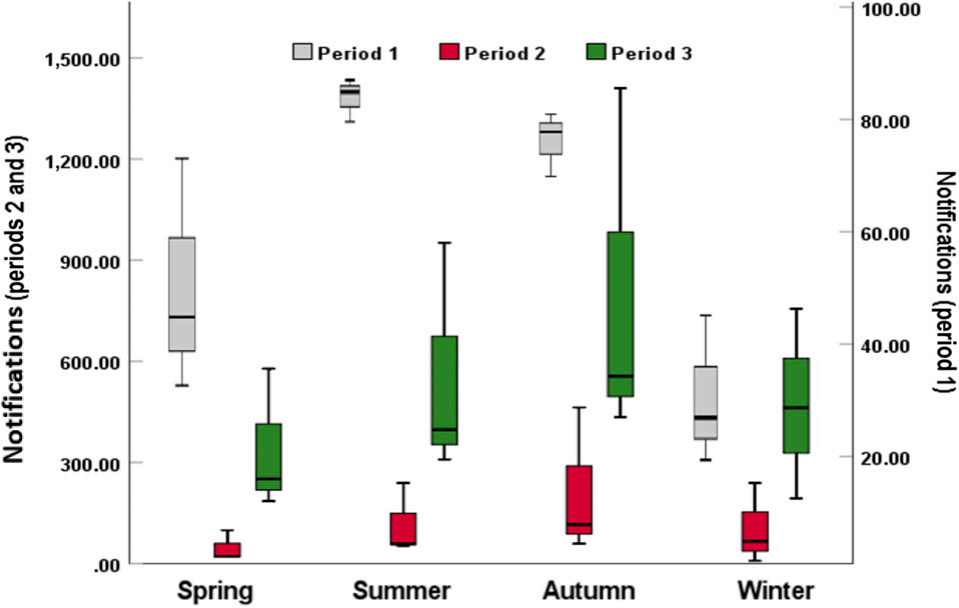
Boxplots of pertussis notifications by season at the Shandong provincial level, 2009–2017. This figure appears in color at www.ajtmh.org.

**Figure 4.**
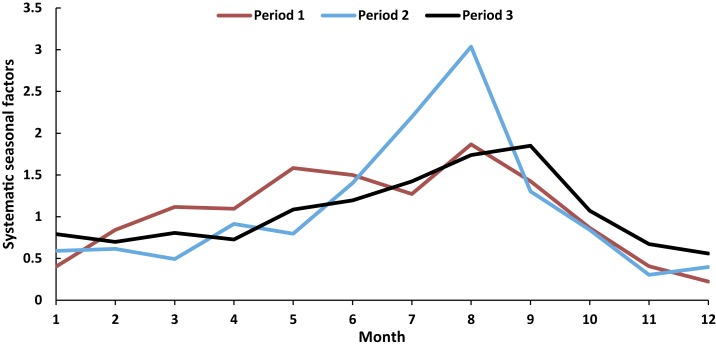
Systematic seasonal variations of pertussis occurrence by month in Shandong Province, 2009–2017. This figure appears in color at www.ajtmh.org.

The pertussis occurrences in periods 2 and 3 were found to peak in autumn with 212.7 and 800.3 cases, respectively (Figure 3). Interestingly, the peak of pertussis was observed in summer in period 1, with the value of 85.0 in the province (Figure 3). Furthermore, there were generally increasing temporal trends in the pertussis infections in summer (rising from 85.0 in period 1 to 552.7 in period 3), autumn (rising from 75.0 in period 1 to 800.3 in period 3), and winter (rising from 28.3 in period 1 to 470.3 in period 3). However, the notifications in spring declined from 51.7 in period 1 to 46.7 in period 2, and then climbed to 338.0 in period 3. Moreover, clear seasonal patterns were evident over the study period. Pertussis infections were observed to peak in August in periods 1 and 2 (Figure 4), but moved to September in period 3 (Figure 4).

**Figure 5.**
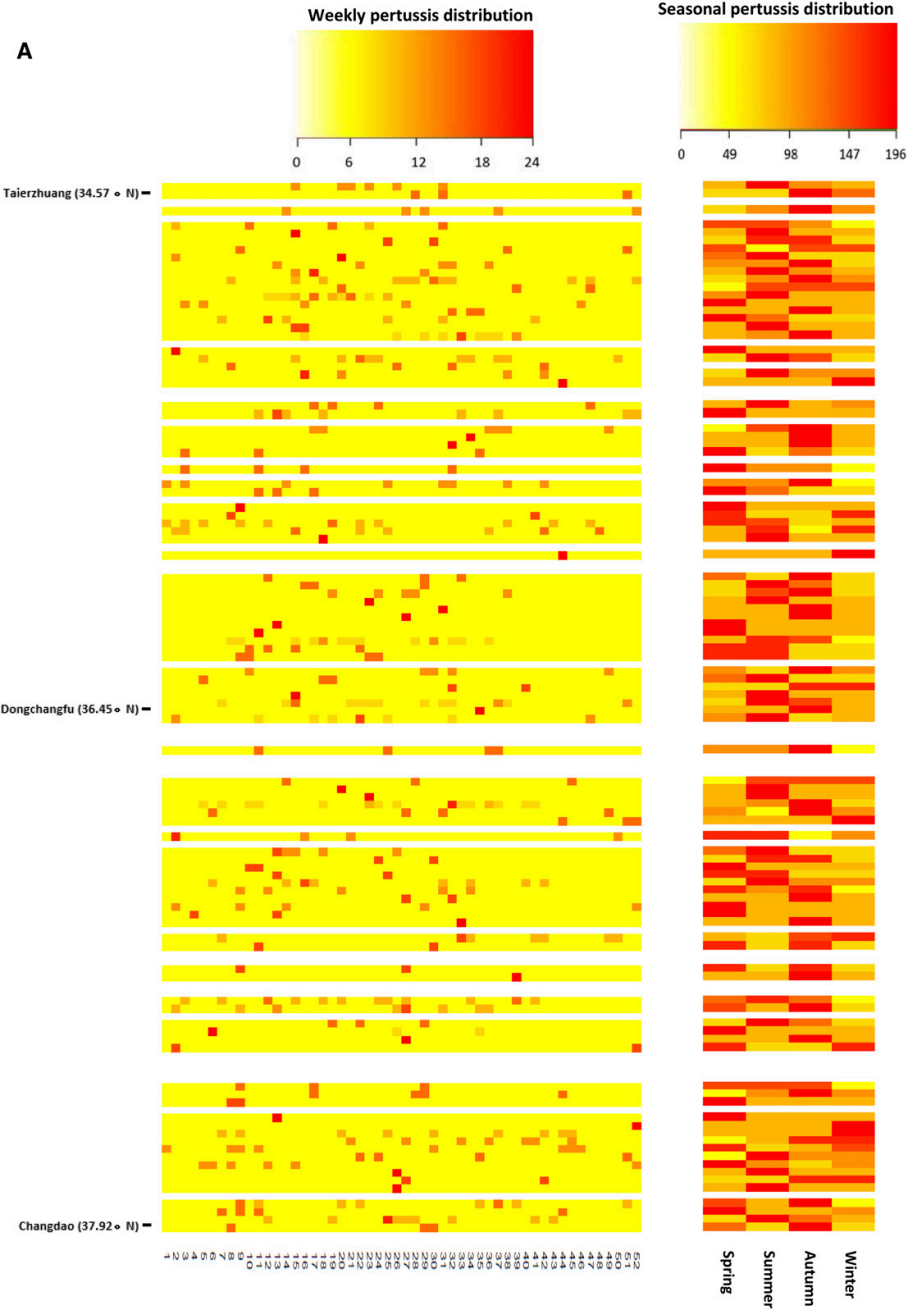

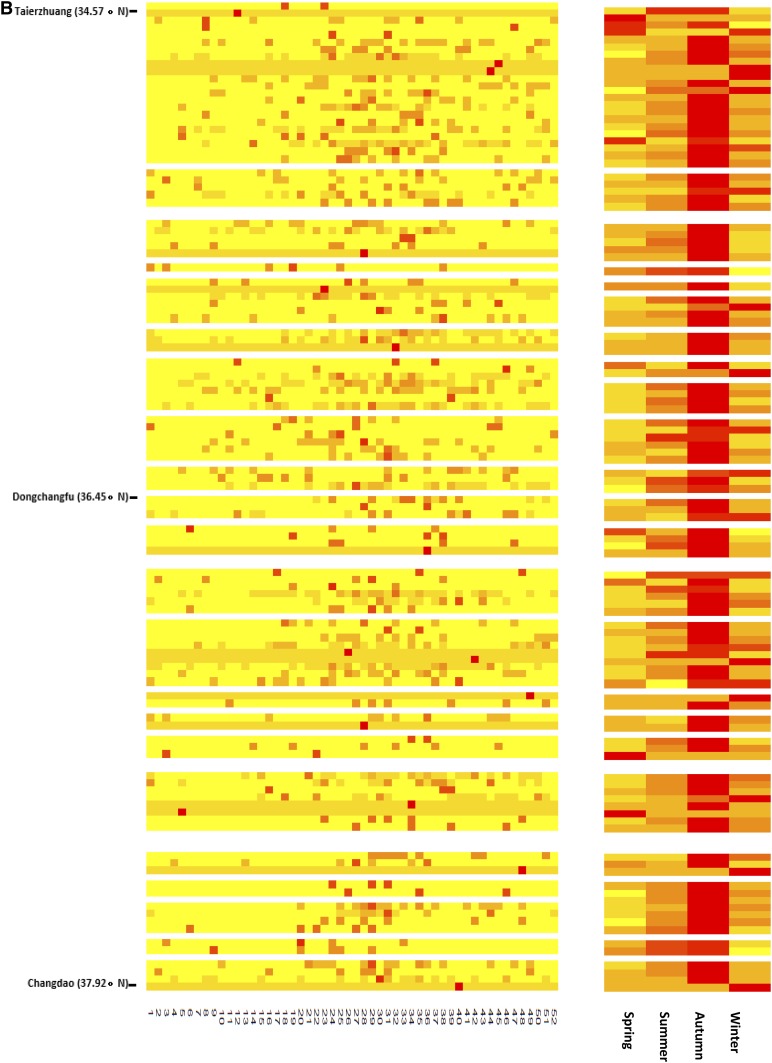

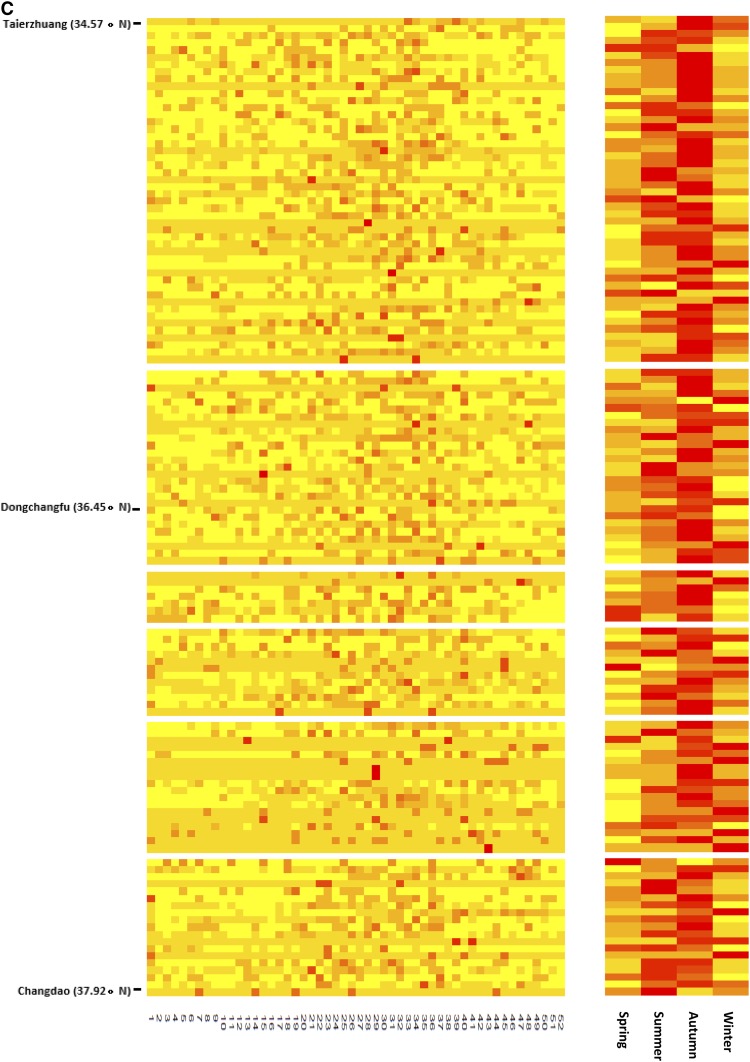
Heat maps of pertussis occurrence in Shandong Province at the county level, by week (left panel) and season (right panel), 2009–2017 (**A**: period 1, **B**: period 2, **C**: period 3; and blank row: no pertussis occurred in the county). This figure appears in color at www.ajtmh.org.

The heat maps showed that most counties experienced strong annual autumn seasonality of pertussis infections in period 2 and period 3 (Figure 5B and C). The seasonality of pertussis in period 1 was not as clear as in other periods (Figure 5A). Furthermore, a comparison of Figure 4A–C, showed that there was an increasing trend in coincidence of peak times (autumn) in pertussis occurrences over the study period.

### The spatial variation and clusters of infection risk.

**Figure 6.**
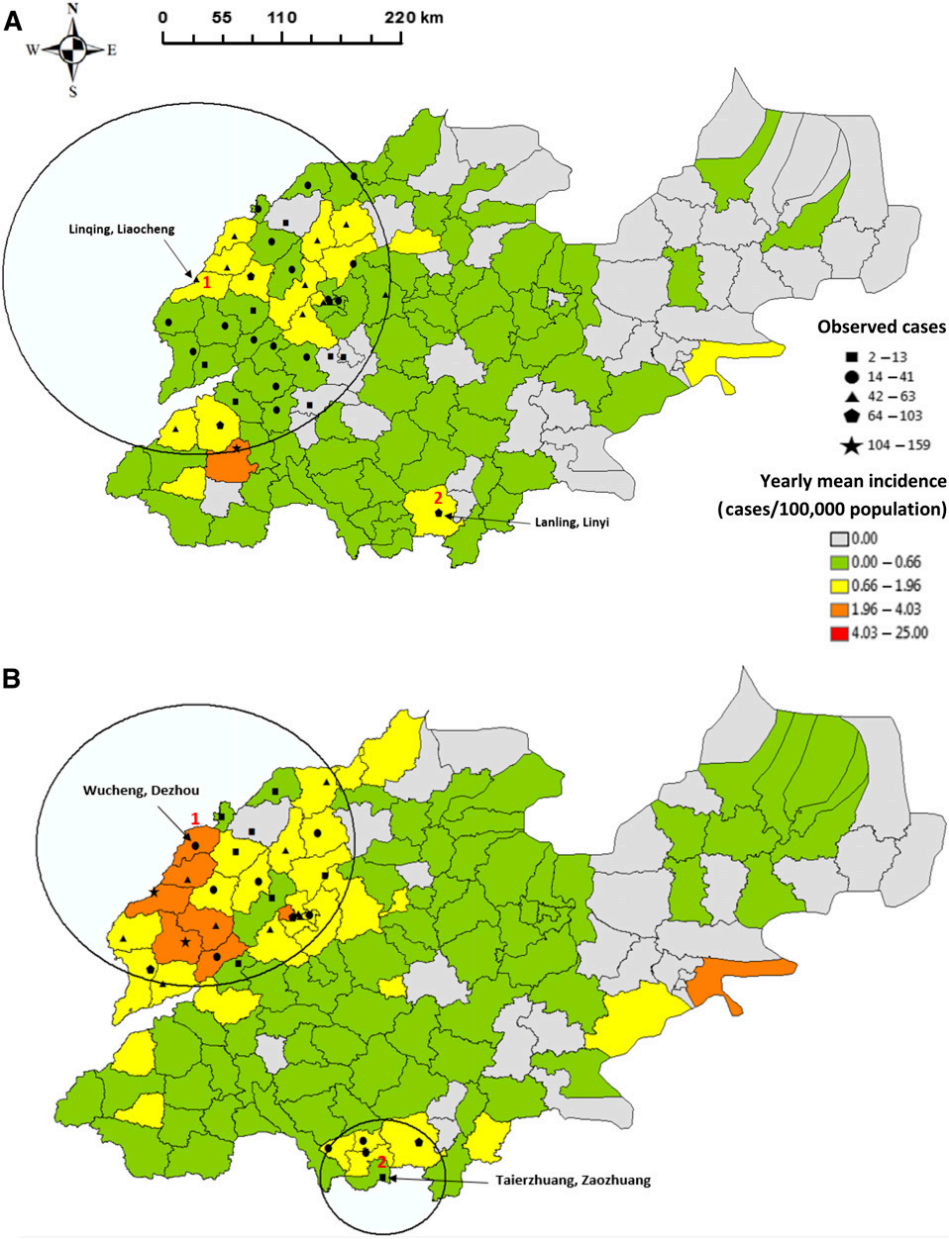

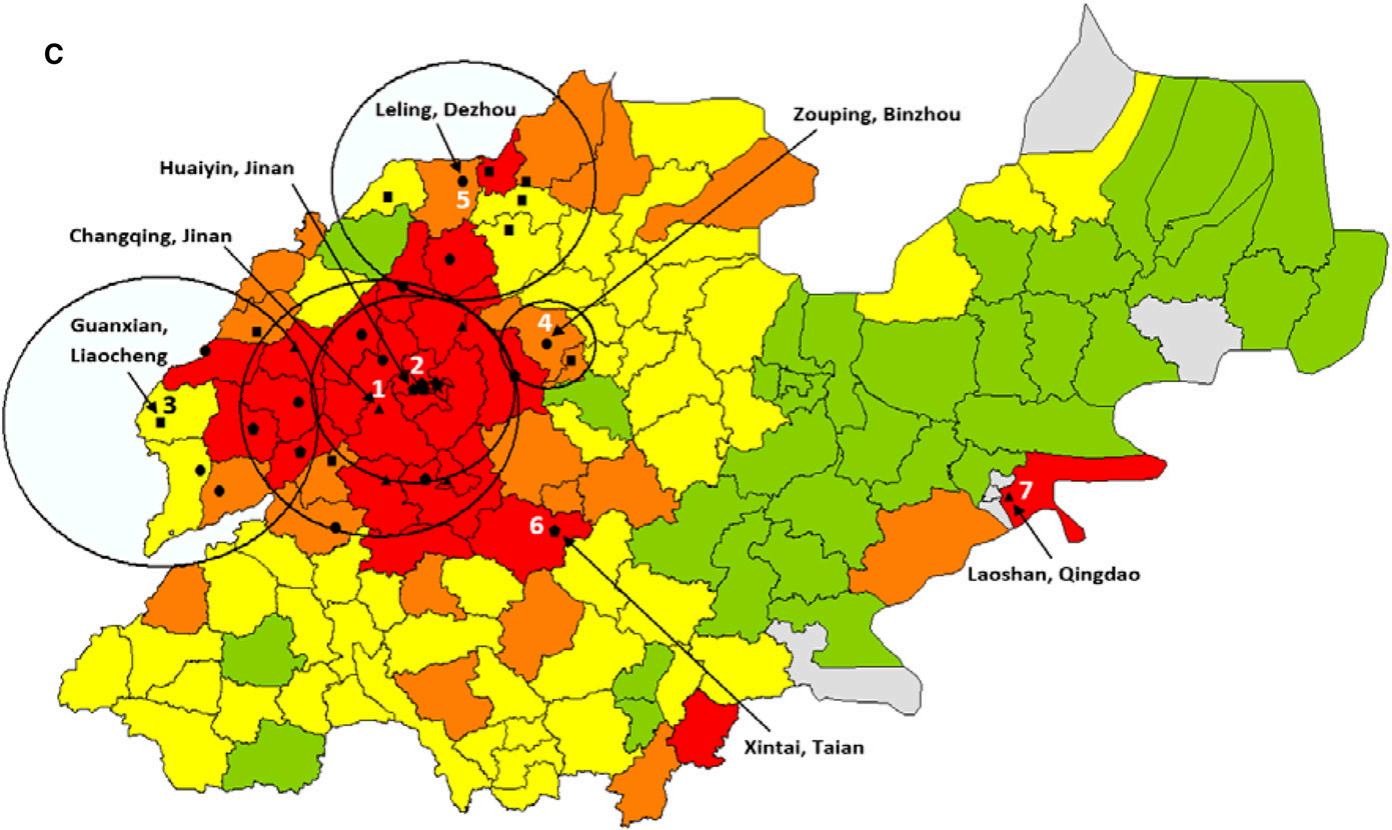
Spatial distributions of incidence and clusters of pertussis infection across Shandong Province, 2009–2017 (**A**: period 1, **B**: period 2, and **C**: period 3). This figure appears in color at www.ajtmh.org.

The average yearly county-specific pertussis incidence was 0.3/100,000 population (range: 0–2.4/100,000 population) for period 1 (2009–2011). SaTScan analysis detected two significant clusters of higher than expected infection rates between 2009 and 2011 (Figure 6A), including one centered in Linqing, Liaocheng (cluster 1, radius: 166.9 km), and one centered in Lanling, Linyi (cluster 2), involving Jinan, the northwestern and southern areas of Shandong Province. Clusters 1 and 2 attributed a risk of infection of 3.8 (95% CI: 3.0–4.9) and 4.1 (95% CI: 2.3–7.4) times higher than other areas, respectively. Moreover, the average yearly county-specific incidence in period 2 was 0.5/100,000 population (range: 0–3.7/100,000 population) with two significant clusters (Figure 6B), which were centered in Wucheng, Dezhou (cluster 1, radius: 124.9 km), and Taierzhuang, Zaozhuang (cluster 2, radius: 50.8 km). These high-risk areas were similar in locations to those identified for period 1. The RR estimates for clusters 1 and 2 were 5.7 (95% CI: 4.8–6.8) and 2.9 (95% CI: 2.1–4.1), respectively.

A total of seven significant clusters of higher than expected infection rates were found in period 3, with a mean yearly county-specific incidence rate of 2.4/100,000 population (range: 0–2.4/100,000 population) (Figure 6C). This included five clusters located in the northwestern areas, including Changqing, Jinan; Huaiyin, Jinan; Guanxian, Liaocheng; Zouping, Binzhou; and Leling, Dezhou (clusters 1–5), and another two clusters at Xintai, Taian (cluster 6), and Laoshan, Qingdao (cluster 7). The highest risk area was centered in Changqing, Jinan (cluster 1, radius: 73.8 km), with a RR of 6.1 (95% CI: 5.6–6.7); the estimated RR of infection for the other clusters ranged from 1.5 to 5.4. Details of the spatial clusters for each period are shown in Table 1.

**Table 1:** Details of spatial clusters of pertussis infection in Shandong Province, 2009–2017

	Cluster (center)	Radius (km)	Number of counties involved	Number of observed cases	Number of expected cases	RR (95% CI)	Log likelihood ratio	*P*-value
A	1 (Linqing, Liaocheng)	166.9	38	147	67.1	3.8 (3.0–4.9)	55.4	< 0.001
	2 (Lanling, Linyi)	0	1	12	3.0	4.1 (2.3–7.4)	7.7	0.02
B	1 (Wucheng, Dezhou)	124.9	27	255	80.0	5.7 (4.8–6.8)	166.8	< 0.001
	2 (Taierzhuang, Zaozhuang)	50.8	5	38	13.6	2.9 (2.1–4.1)	15.3	< 0.001
C	1 (Changqing, Jinan)	73.8	20	1,068	297.54	6.1 (5.6–6.7)	782.3	< 0.001
	2 (Huaiyin, Jinan)	53.9	11	684	172.1	5.4 (4.9–5.9)	504.6	< 0.001
	3 (Guanxian, Liaocheng)	83.4	9	319	132.2	2.7 (2.4–3.0)	103.2	< 0.001
	4 (Zouping, Binzhou)	25.2	3	90	48.2	1.9 (1.5–2.4)	14.8	< 0.001
	5 (Leling, Dezhou)	68.9	8	123	85.2	1.5 (1.2–1.8)	7.7	0.04
	6 (Xintai, Taian)	0	1	93	28.8	3.3 (2.7–4.1)	45.8	< 0.001
	7 (Laoshan, Qingdao)	0	1	37	8.3	4.5 (3.3–6.3)	26.8	< 0.001

A: period 1, B: period 2, and C: period 3.

Furthermore, there were strong spatial and temporal variations in the RR of pertussis infections across Shandong Province at the county level. The areas with high RR values were mainly located in the northwest and south Shandong. However, period 3 had the largest range of RR values across the study time (Supplemental Figure 1D), followed by period 1 (Supplemental Figure 1B), period 2009–2017 (Supplemental Figure 1A), and period 2 (0.00–8.09) (Supplemental Figure 1C).

### Temporal and geographic variations in epidemic dynamics.

**Figure 7.**
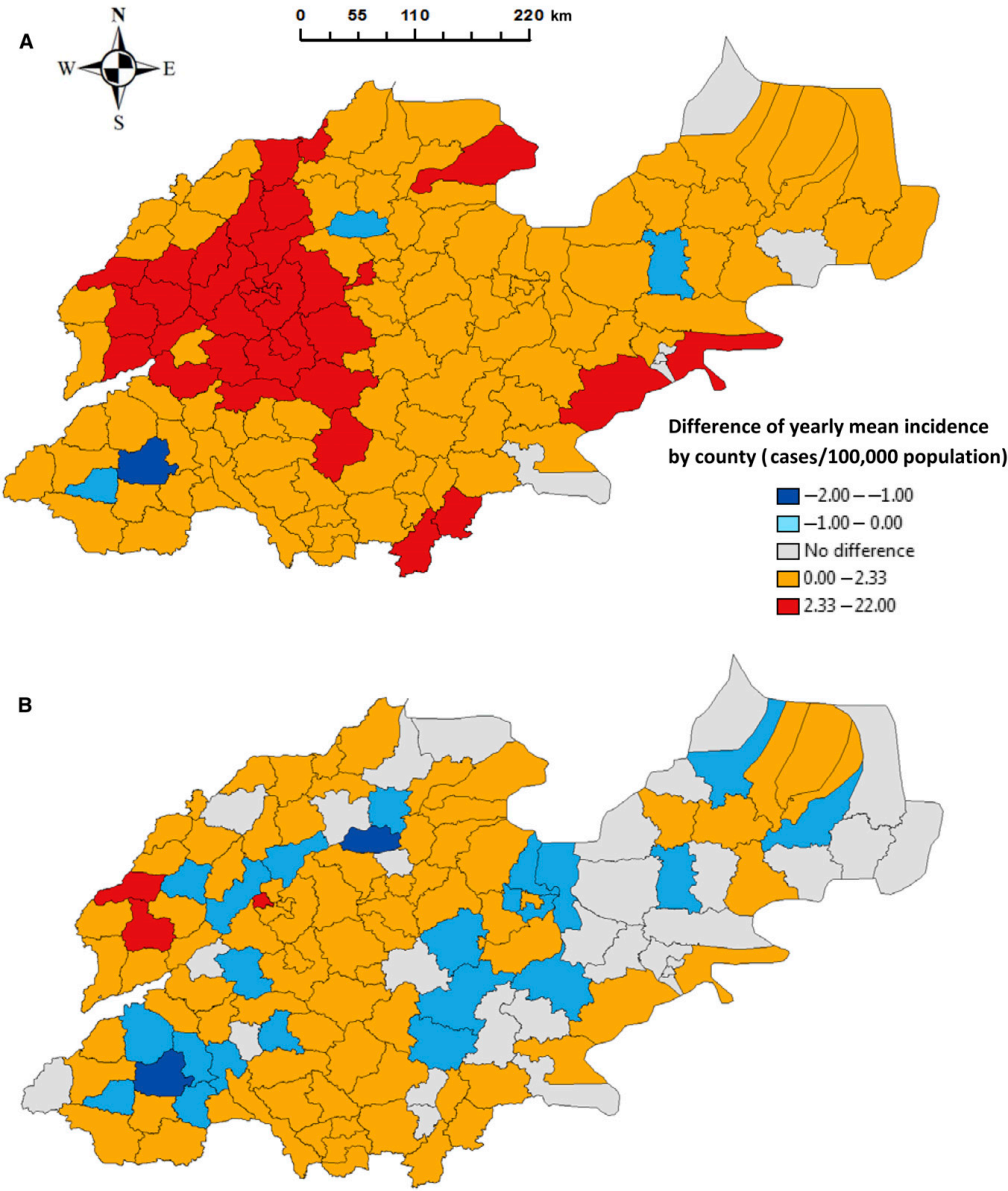

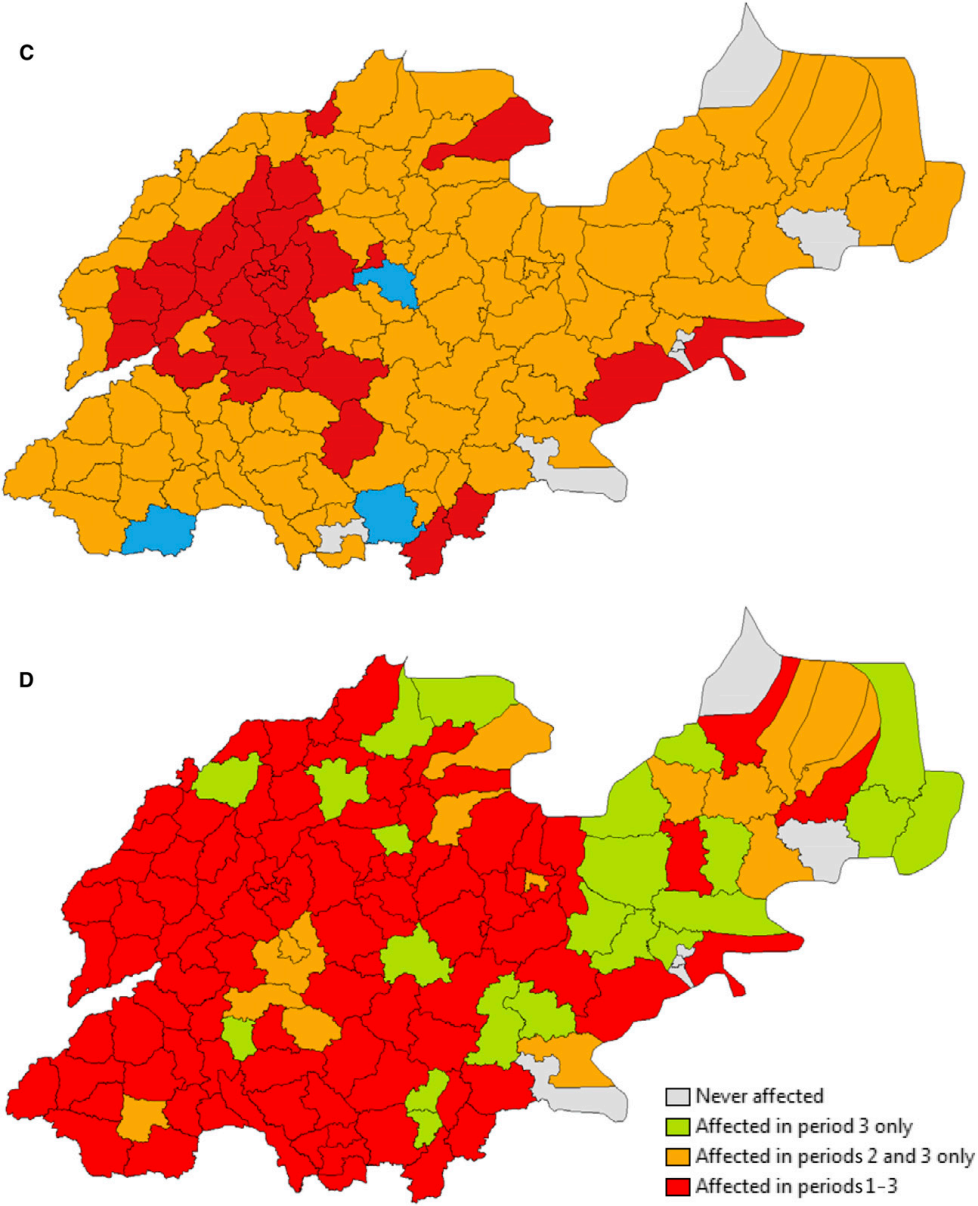
Spatial distribution of the difference in pertussis infection and affected counties, Shandong Province, 2009–2017 (**A**: difference between period 1 and 3, **B**: difference between period 1 and 2, **C**: difference between period 2 and 3, **D**: affected counties in each period). This figure appears in color at www.ajtmh.org.

The mean difference in yearly county-specific pertussis incidence rates was 2.1/100,000 population (range: −1.9 to 21.7/100,000 population) in Shandong Province between periods 1 and 3. Increases in pertussis incidence occurred in all but four counties of Shandong Province during the study period (Figure 7A). Most dramatic increases in incidence occurred in the northwestern and southern parts of Shandong Province.

The mean difference in yearly county-specific incidence rates between periods 1 and 2 was 0.3/100,000 population (range: −1.9 to 3.0/100,000 population). It was noted that 24 of the 137 counties in the province had reduced incidence rates between the two periods (Figure 7B). However, most of these declines occurred between periods 1 and 2, with only three counties experiencing a decline between periods 2 and 3 (Figure 7C). The mean value of the difference was 1.8/100,000 population (range: −0.3 to 20.0/100,000 population) in the province between these last two periods. Similarly, most of the dramatic increases in incidence were observed in the northwestern and southern parts of the province.

Interestingly, opposite trends in incidence were observed in a number of counties when the study period was divided into three subsets, with most of the affected areas located in the middle and western parts of the province. However, closer inspection revealed that these counties had decreasing trends in incidence between periods 1 and 2, but then experienced a rise in infections between periods 2 and 3. Moreover, there was an increasing trend in the number of counties, which had increased incidences between each period, from 83 (between periods 1 and 2) to 124 (between periods 2 and 3). Three counties experienced reduced incidences between periods 2 and 3, but increased incidences between periods 1 and 2 (Figure 7C). Moreover, the spatial patterns of pertussis occurrence showed an obvious geographic expansion from these previous hot spots of persistence over the study period (Figure 7D).

### The directions of pertussis infection dynamics.

The logistic analyses of the positive or negative differences in pertussis incidence between the study periods, using the locations of counties as predictor variables, revealed the following. First, a 1°C change in the longitude to west was associated with a 52% increase in the odds of a positive difference (Table 2). Similarly, the odds increased by 52% and 49% when the longitudes changed 1°C to west for periods 1–2 and periods 2–3 separately. Moreover, the odds of new observation of pertussis, which only affected in periods 2 and 3, increased 105% and 66% when the longitude and latitude changed 1°C to east and north, respectively. The odds of observation in the counties which only affected in period 3 climbed to 132% and 69% when the longitude and latitude changed 1°C to east and north separately.

**Table 2:** Logistic regression of the differences in pertussis incidences between periods and the geographic expansion of infections on the locations of counties in Shandong Province, 2009–2017

	Odds ratio (95% CI; *P*-value)	
	Longitude	Latitude
A	1.52 (1.05–2.17; 0.03)	1.14 (0.55–2.34; 0.73)
B	1.52 (1.20–1.89; < 0.001)	0.85 (0.58–1.26; 0.42)
C	1.49 (1.04–2.17; 0.03)	1.86 (0.88–3.93; 0.10)
D	2.05 (1.40–3.00; < 0.001)	1.66 (0.86–3.22; 0.04)
E	2.32 (1.63–3.31; < 0.001)	1.69 (1.06–2.99; 0.04)

A: difference between periods 1 and 3, B: difference between periods 1 and 2, C: difference between periods 2 and 3, D: affected in periods 2 and 3 only, and E: affected in period 3 only.

### The regression tree analysis of spatial dynamics in infection.

The regression tree analysis of differences in annual pertussis incidence, using the geographic location of counties as predictors, revealed the following. As indicated in Supplemental Figure 2, 118.0°E was the primary classifying factor in all of the models. The mean difference in yearly county-specific pertussis incidence between periods 1 and 3 increased by more than 4-fold (8.6/2.1) for counties with longitude less than 118.0°E and latitude between 36.3°N and 36.8°N (Supplemental Figure 2A). Similarly, the corresponding mean difference increased by more than 4-fold (7.3/1.8) between periods 2 and 3 (Supplemental Figure 2C), and by 5-fold (1.5/0.3) between periods 1 and 2 for the counties with longitudes less than 118.0°E and latitudes between 36.2°N and 36.7°N (Supplemental Figure 2B).

## DISCUSSION

Our results showed that the mean weekly notifications dramatically increased by 7.5 times over the study period, from 4.9 cases in 2009–2011 to 41.6 cases in 2015–2017. Previous studies indicate that the appearance of erythromycin-resistant *B. pertussis* and evolution of *B. pertussis* might be the causes for increase in the occurrence of pertussis in China.^35–37^ Other potential factors that may lead to increasing pertussis epidemics, such as the changes in the type of vaccine and diagnostic test, have been investigated in some developed countries^38,39^; however, these factors have not been examined in China. Further studies are needed to discover the potential reasons for the increasing trend in pertussis infection in China.

The decomposition analysis of pertussis notifications showed clear seasonal patterns at both provincial and county levels. Larger numbers of pertussis infections were observed during autumn in the study period. This finding is supported by a study in Australia, which found that pertussis occurrences peaked in autumn months.^15^ Interestingly, changing seasonality was found when we divided the study period into subsets. An obvious summer seasonality of infection was observed in period 1, but shifted to a strong autumn seasonality in periods 2 and 3. These results are in agreement with previous studies, which reported that the seasonal profile of pertussis occurrence can change in the same area for different time periods.^16,17^ This is difficult to explain, but might indicate important factors in the natural history of pertussis^17^ or changes in exposure or management of the disease. Moreover, the seasonal pattern at the county level (heat map) for period 1 was not as clear as that at the provincial level (boxplots), which showed an obvious summer seasonality in pertussis. The reason might be because period 1 had the least pertussis occurrences over the study period. This led to a difficulty in identifying the peaks in diseases epidemics,^40^ as a small number of infections were distributed over more than 100 counties. We hope to explore the reasons for the seasonal dynamics of pertussis in Shandong Province in future work.

Spatial analysis in our study demonstrated a shift of high-risk areas of pertussis infection over time. The areas with the largest RR values were located in the south of the province (Lanling, Linyi) in period 1, but in the northern part of the province (Wucheng, Dezhou, and Changqing, Jinan) for periods 2 and 3. This might result from pertussis spreading to the more densely populated areas with dramatic change in patterns of population movement over the study period. This has been seen as an important factor for the recent change of spatial structure in pertussis epidemics in the United States.^41^

Moreover, both the number of significant clusters and the corresponding RR values increased over time in Shandong. There were two significant clusters in periods 2 and 3, but seven clusters were found in period 3. Meanwhile, the largest RR value for a cluster was 4.1 (Lanling, Linyi) for period 1, and this increased to 5.7 (Wucheng, Dezhou) for period 2 and rose to peak in period 3 with a value of 6.1 (Changqing, Jinan). The results revealed that a rising number of counties might have experienced an increased exposure to pertussis infection with a spatial shifting of high-risk areas throughout Shandong during the study period. This will be valuable for government and public health authorities to identify spatial risk areas and to minimize the impact of pertussis epidemics by developing efficient distribution of preventive resources.

Interestingly, we observed directional trends in the differences of incidences and the pertussis spread over the study period. An increase in odds of increased incidences between periods was associated with the change of longitudes to west. This meant that west Shandong had greater risk of increased infection in the period. Furthermore, we found that pertussis infections significantly spread to the east and north sides of the province over time. The results are supported by the findings of previous studies, which highlighted the spatial variation of pertussis epidemics.^41,42^ Hence, this might indicate the changing effectiveness of the prevention or control measures used over the period in different areas. The regression tree models identified that the longitude of 118.0°E was a key determinant of the difference in pertussis incidence between periods. This indicated that the areas that were located west of 118.0°E had relatively higher risk of increased infection over time. The results demonstrated that the models, in general, built on the coordinates of counties provide common spatial threshold values for the locations of potential risk areas of pertussis activity using official pertussis data reported by health authorities.

It should be noted that the northwestern part of Shandong Province, which includes the capital city, Jinan, played an important role in the temporal and spatial dynamics of pertussis transmission. First, the highest incidence generally was observed in the area during 2009–2017 and each subset of the period. Second, most of the significant clusters with large RR values were observed in the area, especially in Jinan. Finally, the most dramatic increases in pertussis incidence over the study period occurred in Jinan and its surrounding areas. These results revealed that this area was a relatively high-risk area for pertussis infection in Shandong Province. As pertussis transmission dynamics can be driven by population density and socioeconomic level,^43^ the development of high-risk areas of pertussis infection might result from the dramatically increased share of provincial total population in the last decade (from 19.4% to 52.2%)^44^ and relatively higher income level in this area.^45^ In addition, this might also result from the characteristic behavior of pertussis, which generally arrives in urban centers, and then disseminates to surrounding regions.^46^

The combined effect of increased infection, coincident occurrence in time, and expansion of the geographic range of pertussis brings serious public health concerns. First, resurgent and repeated infection of pertussis could enhance the chances of mutation and/or recombination of the pathogen, which might contribute to an increased transmission among humans.^47,48^ Second, the obvious coincidence of infection in time in different locations might bring pertussis infection peaks toward each other between counties, especially in autumn. As a result, the development of coinfection would occur because of the easy dissemination between humans. Finally, as the extent of the expansion of the spatial range of pertussis infection has not been fully examined, this resurgent disease might spread further within the province and China, and even internationally through dramatically increased movement in human population.^49^

Several limitations were identified for this study. First, CNNDRS only includes the reported number of cases, and this database does not include patients who have pertussis but do not seek medical care or have been misdiagnosed by a clinic and laboratory. Second, the increased reporting awareness by the physicians in hospitals and clinics may influence the reported number of cases. Third, the shift to polymerase chain reaction testing may also affect the results.

In conclusion, pertussis presently remains as a resurgent infectious disease globally. This has posed a threat to public health in China over the last decade. The seasonal and spatial analyses presented in this article have revealed the substantial variation in pertussis infections in different counties over time, although the same national immunization program has run throughout the province. We have identified a relatively high-risk dynamical window for pertussis in space and time, which might help public authorities to develop and optimize preventative measures and more effective control programs to minimize the impact of pertussis epidemics by location.

## Supplementary Files

Supplemental material
