# Supplementary material for: Resurgence of Pertussis Infections in Shandong, China: Space-Time Cluster and Trend Analysis
Source: Am J Trop Med Hyg. 2019 Apr 15;100(6):1342–54. doi: 10.4269/ajtmh.19-0013 (PMC6553910; doi:10.4269/ajtmh.19-0013)
Supplement: Supplementary file 1 [file tpmd190013.SD1.pdf]

The following are supplemental materials and will be published online only

Supplementary information

Resurgence of pertussis infections in Shandong, China: Space-time cluster and trend analysis

[illegible]

Yuzhou Zhang, Hilary Bambrick, Kerrie Mengersen, Shilu Tong, Lei Feng, Li Zhang, Guifang Liu, Aiqiang Xu and Wenbiao Hu

[illegible]

School of Public Health and Social Work; Institute of Health and Biomedical Innovation, Queensland University of Technology, Brisbane, Queensland, Australia. (Yuzhou Zhang: yuzhou.zhang@hdr.qut.edu.au; Hilary Bambrick: h.bambrick@qut.edu.au; Shilu Tong: s.tong@qut.edu.au; Wenbiao Hu: w2.hu@qut.edu.au)

School of Mathematical Sciences, Queensland University of Technology, Brisbane, Queensland, Australia. (Kerrie Mengersen: k.mengersen@qut.edu.au)

School of Public Health and Institute of Environment and Human Health, Anhui Medical University, Hefei, Anhui, China. (Shilu Tong: s.tong@ahmu.edu.cn)

Shanghai Children's Medical Centre, Shanghai Jiao-Tong University, Shanghai, China. (Shilu Tong: tongshilu@scmc.com.cn)

Shandong Provincial Centre of Disease Control and Prevention, Jinan, China. (Lei Feng: tammyfenglei@163.com; Li Zhang: ZL9127@163.com; Guifang Liu: liu-guifang@163.com; Aiqiang Xu: aqxuepi@163.com)

&Łßßˇ¹øŁı ˛ŁıÆ#˚°–ª Łß#

Wenbiao Hu, School of Public Health and Social Work; Institute of Health and Biomedical Innovation, Queensland University of Technology, Brisbane, Queensland, Australia. Phone: +61 31385724 Email: w2.hu@qut.edu.au;

Figure 1. Spatial distributions of the relative risks at county level across Shandong province, 2009-2017 (A: study period 2009-2017, B: period 1, C: period 2, D: period 3).

A

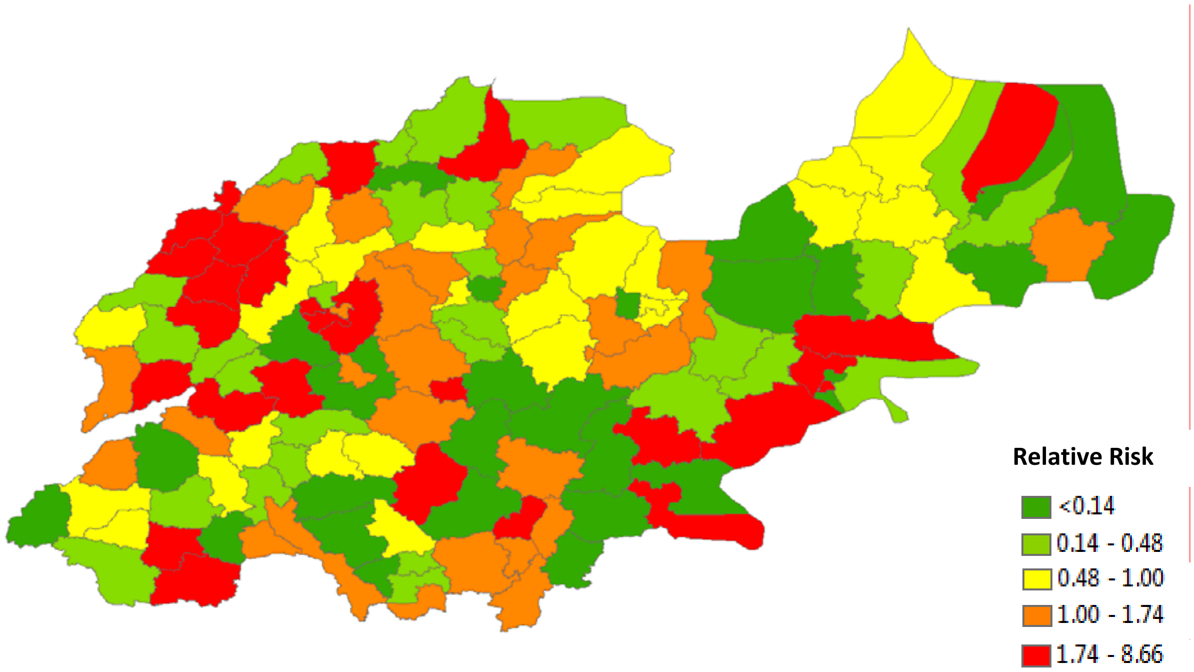

B

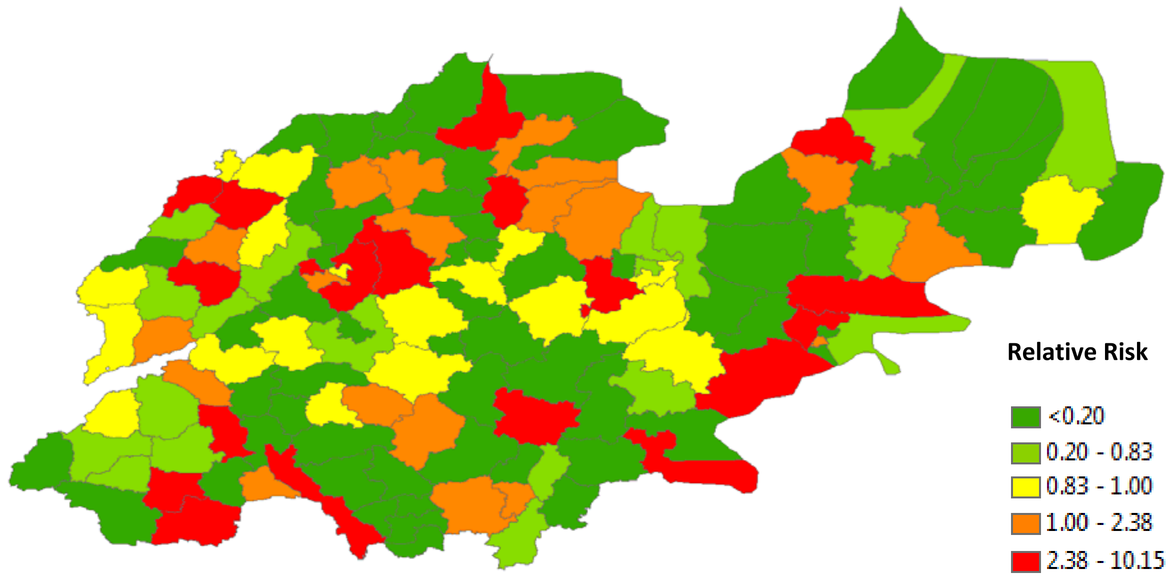

C

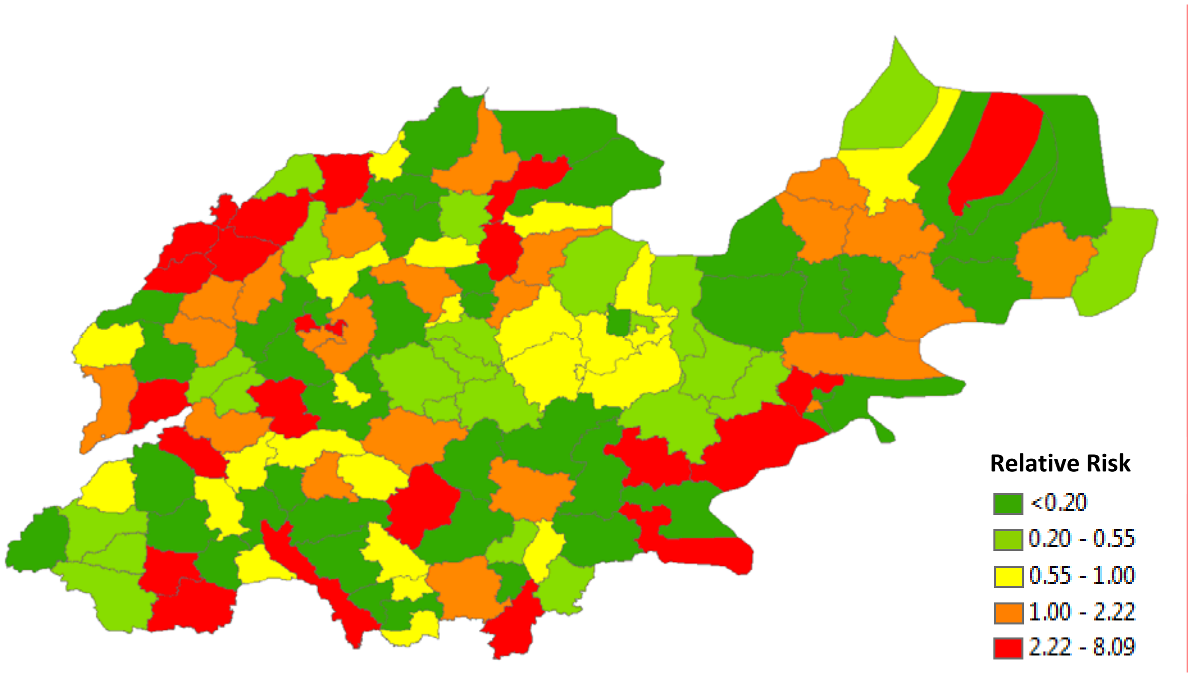
Relative Risk
<0.20
0.20 - 0.55
0.55 - 1.00
1.00 - 2.22
2.22 - 8.09

D

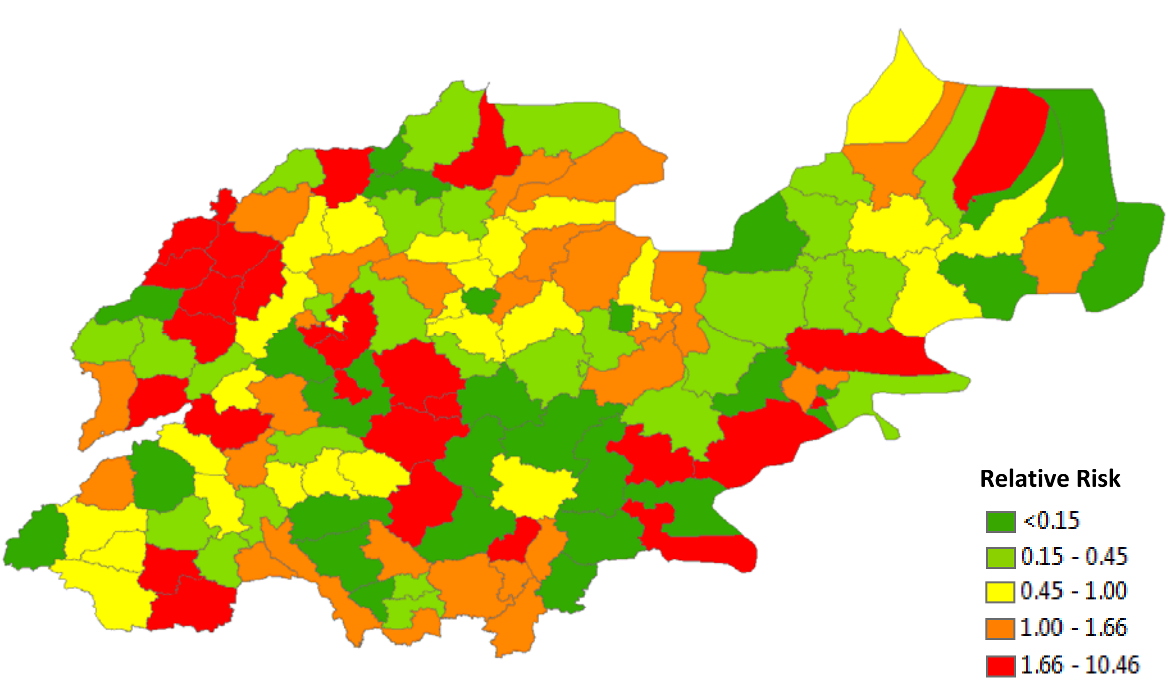
Relative Risk
<0.15
0.15 - 0.45
0.45 - 1.00
1.00 - 1.66
1.66 - 10.46

Figure 2. The regression tree modeling the hierarchical relationship between the differences of yearly mean pertussis incidence between periods with the locations of counties in Shandong, 2009-2017. (The regression tree shows the threshold values, mean differences in yearly average pertussis incidence between periods, N is the total number of counties; A: difference between period 1 and 3, B: difference between period 1 and 2, C: difference between period 2 and 3)

A

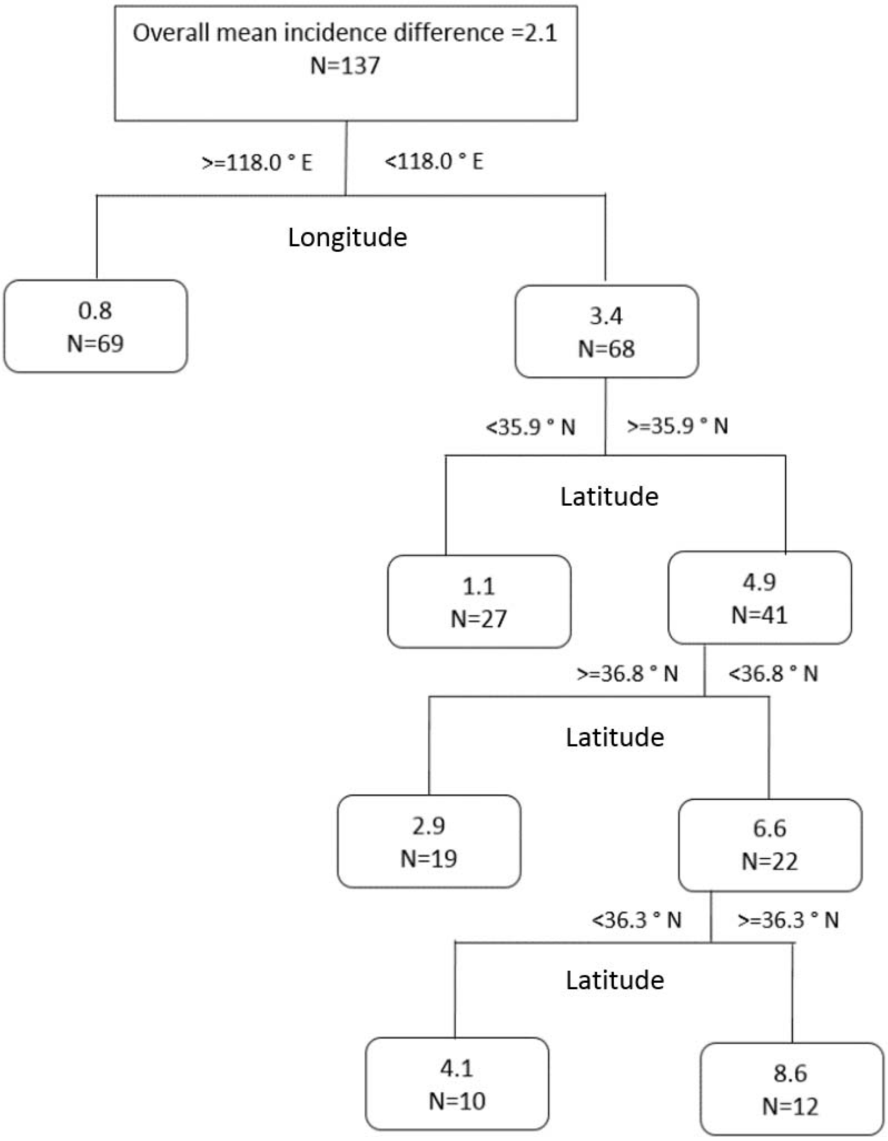

B

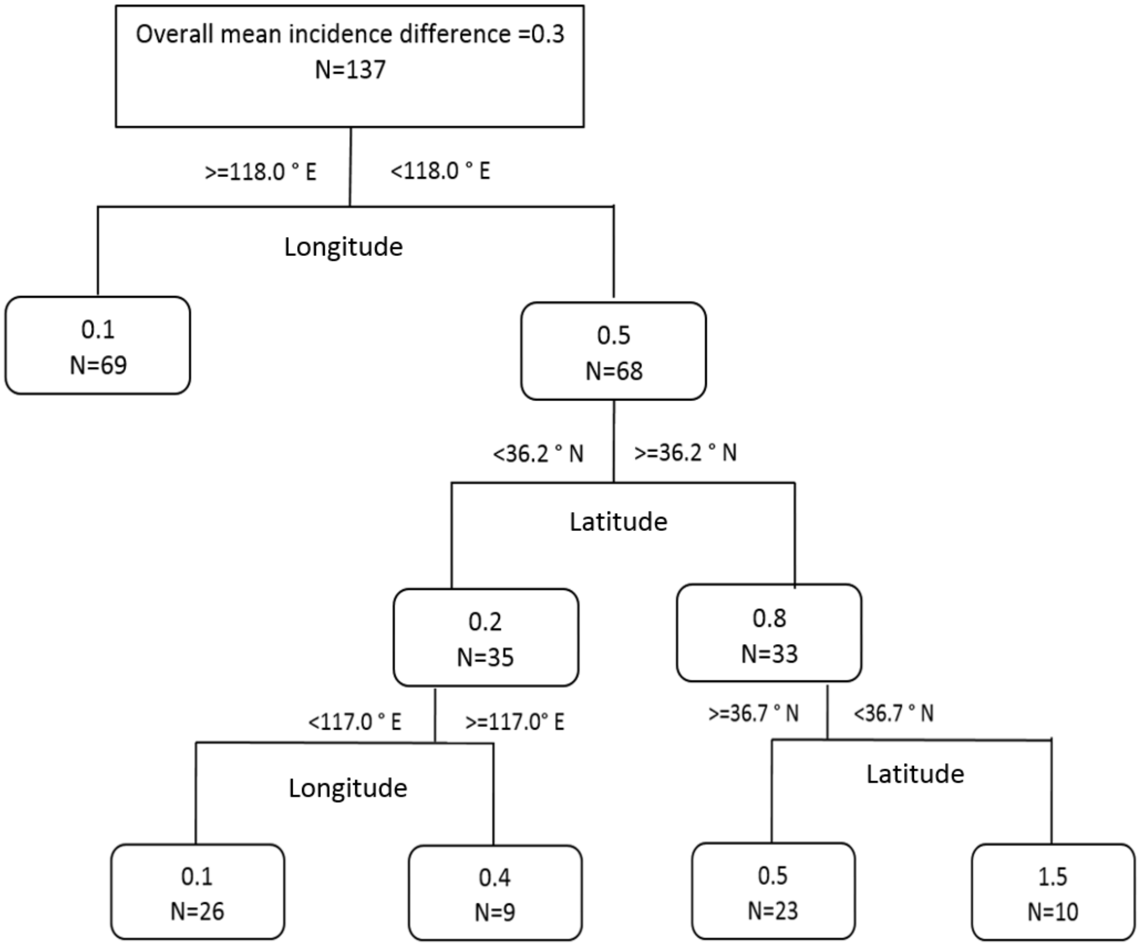
Overall mean incidence difference =0.3
N=137
>=118.0 ° E
<118.0 ° E
Longitude
0.1
N=69
0.5
N=68
<36.2 ° N
>=36.2 ° N
Latitude
0.2
N=35
0.8
N=33
<117.0 ° E
>=117.0° E
>=36.7 ° N
<36.7 ° N
Longitude
Latitude
0.1
N=26
0.4
N=9
0.5
N=23
1.5
N=10

C

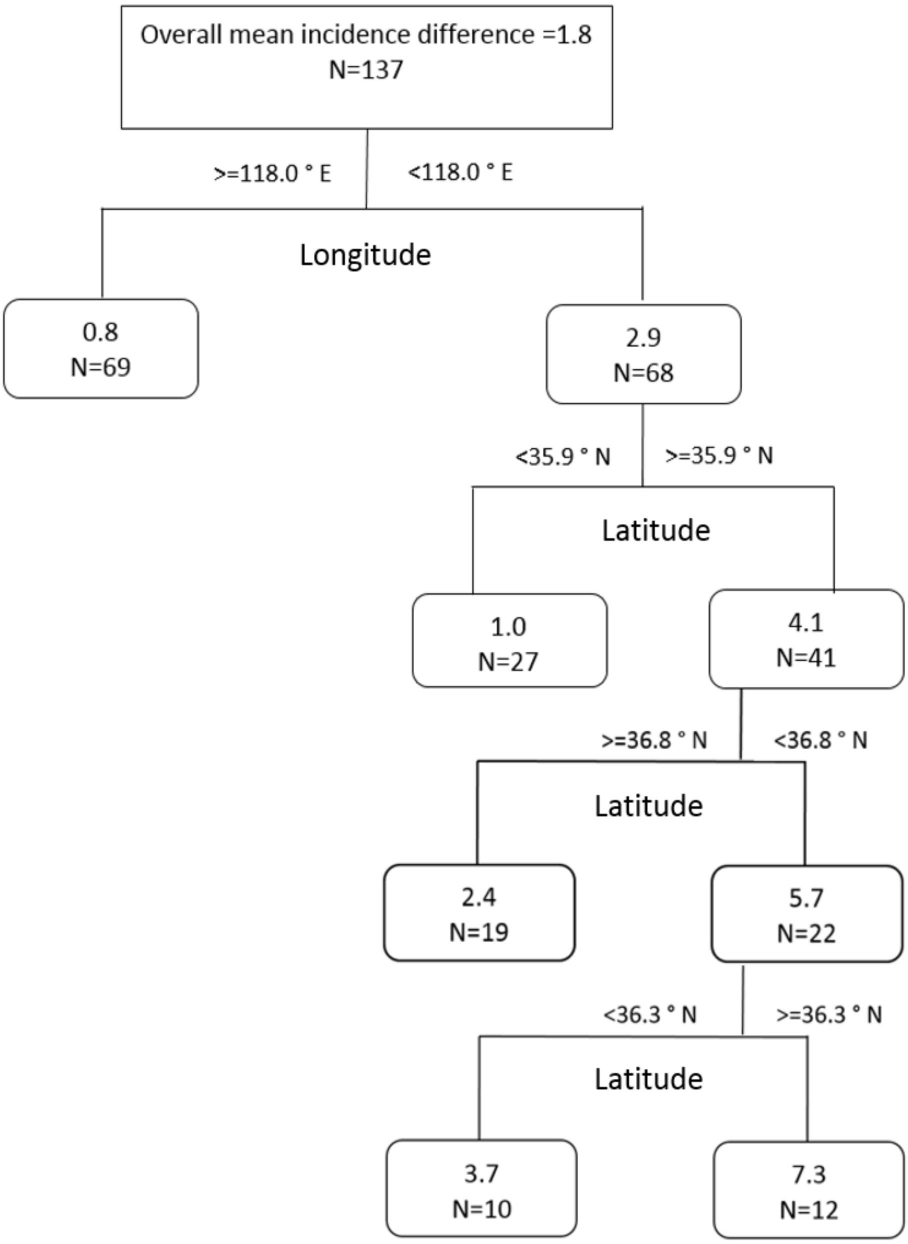

Overall mean incidence difference =1.8
N=137
>=118.0 ° E
<118.0 ° E
Longitude
0.8
N=69
2.9
N=68
<35.9 ° N
>=35.9 ° N
Latitude
1.0
N=27
4.1
N=41
>=36.8 ° N
<36.8 ° N
Latitude
2.4
N=19
5.7
N=22
<36.3 ° N
>=36.3 ° N
Latitude
3.7
N=10
7.3
N=12

The definition of Relative Risk in the study

For Shandong province *A* under study, the RR is defined to be the infection risk λ_Z of county *Z* compared to the risk $\lambda_{A/Z}$ in all other counties *A/Z*. Let

$\lambda_Z=E(Y_Z)/E_Z,$

$E_Z=N*P_Z/P_+,$

where Y_Z is the Poisson random variable of pertussis notifications in county *Z*, with expected cases given by $E(Y_Z)$, E_Z is the observed number of cases in county *Z*, P_Z is the population of county *Z*, P_+ is the total population at risk in the province and *N* is the total number of notifications. Analogously we define $\lambda_{A\backslash Z}$. This way, the RR is defined as $RR=\lambda_Z/\lambda_{A/Z}$.
